# Explainable Prediction of Medical Codes With Knowledge Graphs

**DOI:** 10.3389/fbioe.2020.00867

**Published:** 2020-08-14

**Authors:** Fei Teng, Wei Yang, Li Chen, LuFei Huang, Qiang Xu

**Affiliations:** ^1^School of Information Science and Technology, Southwest Jiaotong University, Chengdu, China; ^2^The Third People's Hospital of Chengdu, Chengdu, China; ^3^School of Information Engineering, Chengdu University of Traditional Chinese Medicine, Chengdu, China

**Keywords:** automated ICD coding, knowledge graphs, explainable, medical records, natural language processing

## Abstract

International Classification of Diseases (ICD) is an authoritative health care classification system of different diseases. It is widely used for disease and health records, assisted medical reimbursement decisions, and collecting morbidity and mortality statistics. The most existing ICD coding models only translate the simple diagnosis descriptions into ICD codes. And it obscures the reasons and details behind specific diagnoses. Besides, the label (code) distribution is uneven. And there is a dependency between labels. Based on the above considerations, the knowledge graph and attention mechanism were expanded into medical code prediction to improve interpretability. In this study, a new method called G_Coder was presented, which mainly consists of Multi-CNN, graph presentation, attentional matching, and adversarial learning. The medical knowledge graph was constructed by extracting entities related to ICD-9 from freebase. Ontology contains 5 entity classes, which are disease, symptom, medicine, surgery, and examination. The result of G_Coder on the MIMIC-III dataset showed that the micro-F1 score is 69.2% surpassing the state of art. The following conclusions can be obtained through the experiment: G_Coder integrates information across medical records using Multi-CNN and embeds knowledge into ICD codes. Adversarial learning is used to generate the adversarial samples to reconcile the writing styles of doctor. With the knowledge graph and attention mechanism, most relevant segments of medical codes can be explained. This suggests that the knowledge graph significantly improves the precision of code prediction and reduces the working pressure of the human coders.

## Introduction

The International Classification of Diseases (ICD) is a standard classification system according to the characteristics of diseases and the rules maintained by the World Health Organization. Each code represents a specific disease, symptom, or surgery. And a set of codes in the medical record represents uniquely diagnostic and procedural information during patient visits. As a significant part of the hospital information system, it is widely used for medical insurance payments, health reports, and mortality calculations. Therefore, the ICD coding task is an essential job in the medical record information department. While ICD codes are important for making clinical and financial decisions, ICD coding is time-consuming, error-prone, and expensive. In most cases, the human coders assign ICD codes to medical records according to the clinical diagnosis record of physician. It is difficult because the code assignment should consider overall the health condition in the long text-free medical records, including symptoms, signs, surgery, medication, body, etc.

Automatic coding uses medical records as input to predict the final ICD codes based on text content. But the automatic ICD coding task usually has the following difficulties: (1) The clinical records of patients are not always structured in the same way. And the vital information in the text is distributed in various segments. For the above two reasons, it is very difficult to extract important and relevant knowledge from various kinds of medical records effectively. (2) Most importantly, the medical field has a lot of terminologies, which is difficult for non-professionals to understand the meaning of these terminologies. Even for the same disease, there are many ways to describe it differently from ICD description. (3) Datasets in the medical field are often small, and doctors have different writing styles. Each physician usually has his way to describe medical terminologies.

In this paper, we proposed a new end-to-end method called G_Coder (Graph-based Coder) for automatic ICD code assignment using clinical records. The contributions of this paper are summarized as follows: (1) We utilize Multi-CNN (multiple convolutional neural networks) to capture local correlation, which extracts key features from the irregular text. (2) We build a knowledge graph, which enriches the meaning of terminologies through integrated related knowledge points. It is combined with the attention mechanism to help understand the meaning of related terminologies, making the coding results interpretable. (3) The adversarial learning is used to generate adversarial samples to increase samples and reconcile the different writing styles.

Our model has outperformed other models in micro-AUC and micro-F1 on MIMIC-III (Multi-parameter Intelligent Monitoring in Intensive Care) datasets with 46 K distinct hospital admissions and top 50 common ICD-9 codes.

## Related Works

### Automatic ICD Coding

It was 20 years ago that many researchers have explored how to automatically assign ICD codes based on clinical records. There are two major categories of approaches for automatically assigning ICD-9 codes using medical records. One category is rule-based and the other category is learning-based. Rule-based systems are manually extracted statistical features by humans. Chen et al. (2017) and Ning et al. (2016) presented an improved approach based on the Longest Common Subsequence (LCS) and semantic similarity for performing ICD-10 code assignment to Chinese diagnoses. But such approaches only consider the simple matching of strings, which is not a medical problem. Beyond that, researchers applied automatic and semi-automatic (Medori and Fairon, 2010) machine learning methods to automatically assign ICD codes. Automatic ICD-9-CM encoding consisted of support vector machines (SVM) (Yan et al., 2010; Adler et al., 2011; Ferrão et al., 2013; Wang et al., 2017), k-nearest neighbors (Ruch et al., 2008; Erraguntla et al., 2012), Naive Bayes (Pakhomov et al., 2006; Medori and Fairon, 2010), and other methods such as topic model (Ping et al., 2010; Perotte et al., 2013). Semi-automatic methods generally require more manual participation and may require manual data processing, feature selection, data verification, etc. Automatic methods generally use a series of operations in an end-to-end manner. Nevertheless, the development of automatic coding technology is not yet mature, and manual verification is inevitable. All the above methods only utilize the statistical characteristics of words and ignore the contextual meaning.

In recent years, many new methods are emerging with the development of deep neural network. Li et al. (2018) combined the convolutional neural network (CNN) and the “Document to Vector” technique to extract textual features. It solves the characteristics of CNN's indistinguishable word order while taking all the words into account. Baumel et al. (2017) applied a hierarchical approach which is Hierarchical Attention bidirectional Gated Recurrent Unit (HA-GRU) to tag a discharge summary by identifying the relevant sentences. It utilizes the Gated Recurrent Unit to encode text, which experimental effect is similar to long short-term memory networks (LSTM), but it is easier to calculate. Yu Y. et al. (2019) explored character features and word features based on bidirectional LSTM with attention mechanism and Xie and Xing (2018) applied tree LSTM with ICD hierarchy information for automatic ICD coding. Compared with ordinary LSTM, bidirectional LSTMs tend to have higher accuracy, and tree LSTM is more suitable for data that is a tree-like hierarchical structure. Mullenbach et al. (2018) proposed to extract per-code textual features across the document using a convolutional neural network and used an attention mechanism to select the most relevant segments for each possible code. Based on that, Li and Yu (2019) combined multi-filter convolutional layers and residual convolutional layers to enlarge the receptive field.

Deep learning methods improved the ability to capture semantic information but ignored the importance of medical knowledge and experience. In practical work, the human coders fully utilize the basic medical knowledge to provide decision support for the work. However, all the methods just mentioned are data-driven approaches or simple mapping, which lack of the theoretical support and suffer from the complicated preprocessing of the noisy text. To build a more explainable ICD coding system, we utilize the knowledge graph as supplementary knowledge to add to the model, which is equivalent to combining a data-driven approach with medical knowledge. What is more, we successively perform text preprocessing and Multi-CNN algorithm to extract text features to reduce text noise. Adversarial learning generates adversarial samples for training to reconcile the different writing styles. The attention mechanism selects the most relevant segments for each possible code.

### Graph Embedding

Graph embedding technology expresses nodes in the form of low-dimensional dense vectors, which require similar nodes in the original graph to be similar in the low-dimensional expression space. The representative work of Graph Embedding is DeepWalk (Perozzi et al., 2014), LINE (Tang et al., 2015), Node2Vec (Grover and Leskovec, 2016), SDNE (Wang et al., 2016), and Struc2Vec (Ribeiro et al., 2017). The obtained expression vectors can be used for downstream tasks, such as node classification (Ye et al., 2018; Gong and Ai, 2019), link prediction (Li et al., 2019a), or visualization (Liu et al., 2020). In the field of biomedicine, graphs are often used to predict drug interactions and predict drug target proteins. The knowledge graph embedding is used to calculate several similarity measures between all drugs in the scalable and distributed framework to obtain the interaction of drugs (Ibrahim et al., 2017). Mohamed et al. (2020) used knowledge graph embeddings to learn the vector representation of all drugs and targets to discover protein drug targets.

### Attention Mechanism

The attention mechanism was first used for machine translation (Dzmitry et al., 2014). It calculates the attention weight of each word in the encoder sequence to each word in the decoder sequence to focus more on the most relevant part of the current word. The attention mechanism improves the effect and also increases the interpretability of the neural network. After adding attention, the weight of the data can be visualized to confirm the correctness of the method. Besides, attention mechanism has the ability to capture global features in long texts. The attention mechanism mimics the internal process of biological observation behavior, which is a mechanism that aligns internal experience and external sensation to increase the observation precision of some areas. It has been successfully used in medical tasks. Such as medical imaging (Ozan et al., 2018), clinical text information extraction (Li et al., 2019b; Xu et al., 2019), and DNA-related tasks (Yu W. et al., 2019; Hong et al., 2020).

### Adversarial Learning

Adversarial learning is to make the two networks compete against each other. The generator network continuously captures the probability distribution of the real data in the training set and transforms the input random perturbation into new samples. The discriminator network observes both real and fake data to determine the authenticity of this data. Through repeated confrontation, the capabilities of the generator and discriminator will continue to increase until a balance is reached. Goodfellow et al. (2015) developed a method named FSGM that can effectively calculate the perturbation. They set the perturbation to the maximum value of the loss function along the direction of the gradient. FSGM takes the same step in each direction, and Goodfellow's subsequent FGM (Miyato et al., 2017) is scaled according to specific gradients to obtain better adversarial samples. Adversarial learning improves the robustness of the model through the idea of games. It randomly adds perturbation factors to the input to simulate unknown data to ensure that the model can work stably in any situation. Adversarial learning has been used for privacy protection (Max et al., 2019) of medical records and named entity recognition (Zhao et al., 2019) in clinical texts.

## Materials and Methods

**Figure 1:**
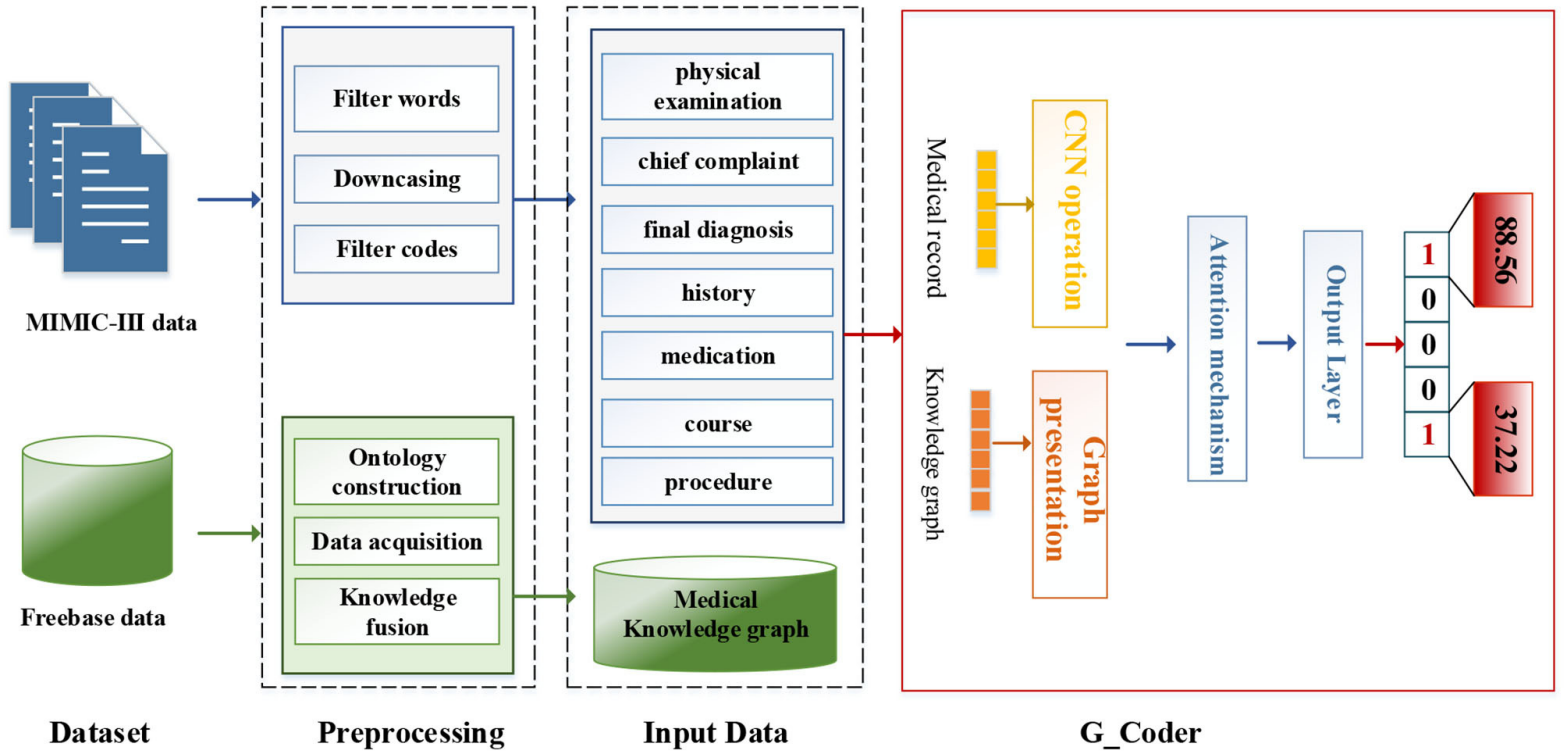
The overall process of this method.

As can be seen from Figure 1, this section will detail all the processes by combining data materials with the proposed methods.

### Dataset and Preprocessing

We utilize the transfer knowledge graph to improve the interpretability and performance of automatic ICD coding. In the study, we select Multi-parameter Intelligent Monitoring in Intensive Care-III (MIMIC-III) dataset (Johnson et al., 2016) as an experimental dataset and Freebase dataset as a source of the knowledge graph. A brief introduction to these two data sets and related preprocessing techniques are as follows.

#### MIMIC-III Dataset

MIMIC-III dataset is the only public database for learning automated ICD-9 coding, which allows fair comparisons with different methods. It contains reliable and comprehensive 58,976 hospital admissions collected between 2001 and 2012 in the Beth Israel Deaconess Medical Center. Each medical record usually includes discharge summaries, survival data, diagnostic codes, vital signs, laboratory measurements, etc. Besides, the discharge summary always contains multiple information, such as “discharge diagnosis,” “past medical history,” physical examination,” and “chief complaint,” etc. Table 1 shows a sample of a medical record in the dataset. The “HADMID” uniquely identifies each medical record. Each hospital admission has a group of ICD-9 codes given by the medical coders. For each medical record, codes distribute unevenly in numbers which varies from one to 39. The number of codes is usually not equal to the number of diagnosis descriptions. It invalidates the one-to-one method of allocating codes. The entire dataset contains 6,984 distinct codes and 943 categories. Each code has a short phrase or a sentence, articulating a disease, symptom, or condition.

**Table 1:** An example of a medical record.

**Medical record (partially shown)**
**HADMID:105501**
Admission Date: [**2172-7-6**] Discharge Date: [**2172-7-10**] Date of Birth: [**2096-4-25**] Sex: M Service: Cardiothoracic Surgery Service HISTORY OF PRESENT ILLNESS: The patient is a 75-year-old gentleman who is a patient of Dr. [**First Name4 (NamePattern1) **] [**Last Name (NamePattern1) 47696**] who was transferred in from [**Hospital3 3583**] status post a myocardial infarction for cardiac catheterization…… PAST MEDICAL HISTORY: 1. Hypertension. 2. Myocardial infarction. 3. Hypercholesterolemia. 4. Myocardial infarction in [**2158**].……
**ICD-9 codes and description**
88.56 Coronary arteriography using two catheters 39.61 Extracorporeal circulation auxiliary to open heart surgery 88.72 Diagnostic ultrasound of heart 36.15 Single internal mammary-coronary artery bypass 584.9 Acute renal failure, unspecified 37.22 Left heart cardiac catheterization 410.71 Acute myocardial infarction, subendocardial infarction, initial episode of care 414.01 Coronary atherosclerosis of native coronary artery 428.0 Congestive heart failure, unspecified 39.95 Hemodialysis

We adopt a series of standard text pre-processing techniques, which contain regular expression matching and tokenization to reduce the noise in raw note texts. Firstly, we extract relevant data from MIMIC-III as input text, which contains “physical examination,” “chief complaint,” “final diagnosis,” “history,” “medication,” “course,” and “procedure.” Secondly, we remove stop words from the input text and transform each token into its lowercase. Simultaneously removing words <3 and replacing unknown words with “UNK.” Thirdly, medical records with associated labels that do not contain the top 50 code are discarded.

#### Freebase

With the rapid development of the knowledge graph in recent years, research-based on knowledge graphs has attracted widespread attention in the medical field. Freebase mainly extracts structured data from wikis and publishes them as RDF. It is fully structured, but the data source is not limited to wikis. It also imports a large number of professional data sets and provides data query and entry mechanisms.

**Figure 2:**
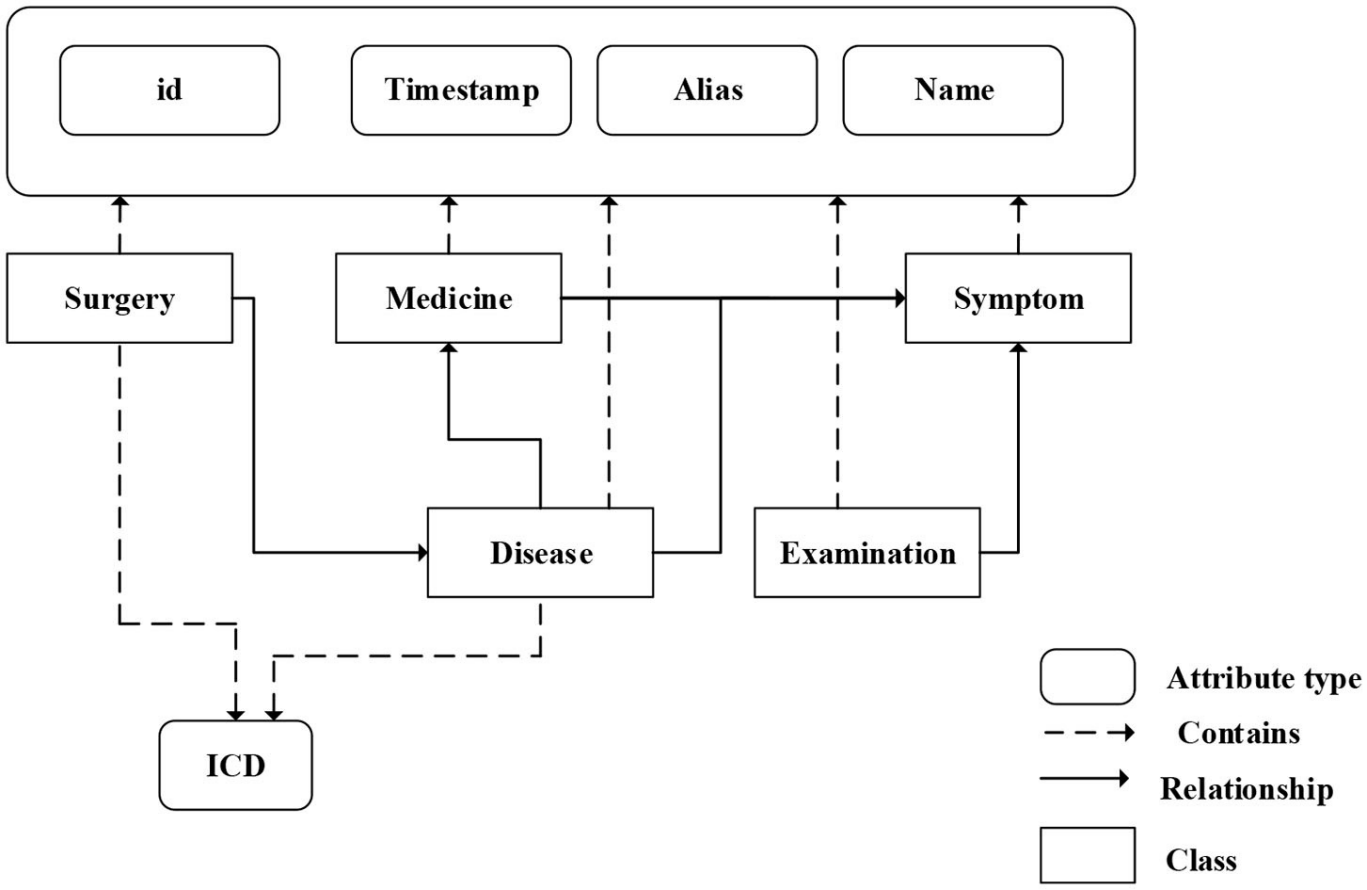
The ontology of medical knowledge graph.

We fuse ICD-9 description information with medical knowledge extracted from freebase to build the final knowledge graph. Freebase Medicine originate from Wikipedia and other datasets such as U.S. National Medical Data. One study has reported that 70% of junior doctors used Wikipedia for health knowledge every week (Trevena, 2011). Because the freebase is reliable, the information provided in Freebase is generally considered to be reliable. The matching method is used for knowledge fusion. Since some diagnosis terms from ICD-9 description imperfectly match Freebase content, we use the ICD description text as the search terms to find the most relevant Freebase content by the Freebase API (http://freebase.gstore-pku.com/). The ontology that was constructed contains 5 entity classes, which are disease, symptom, medicine, surgery, and examination. The constructed ontology is shown in Figure 2, which contains the relationships (disease manifests as symptoms, medicine treats disease, surgery treats disease, and commonly used disease test data, etc.) and attribute types, such as id, name, ICD, etc. In the final knowledge graph, there are 1,560 nodes and more than 20,000 sets of relationships.

### Methods

#### Overview

**Figure 3:**
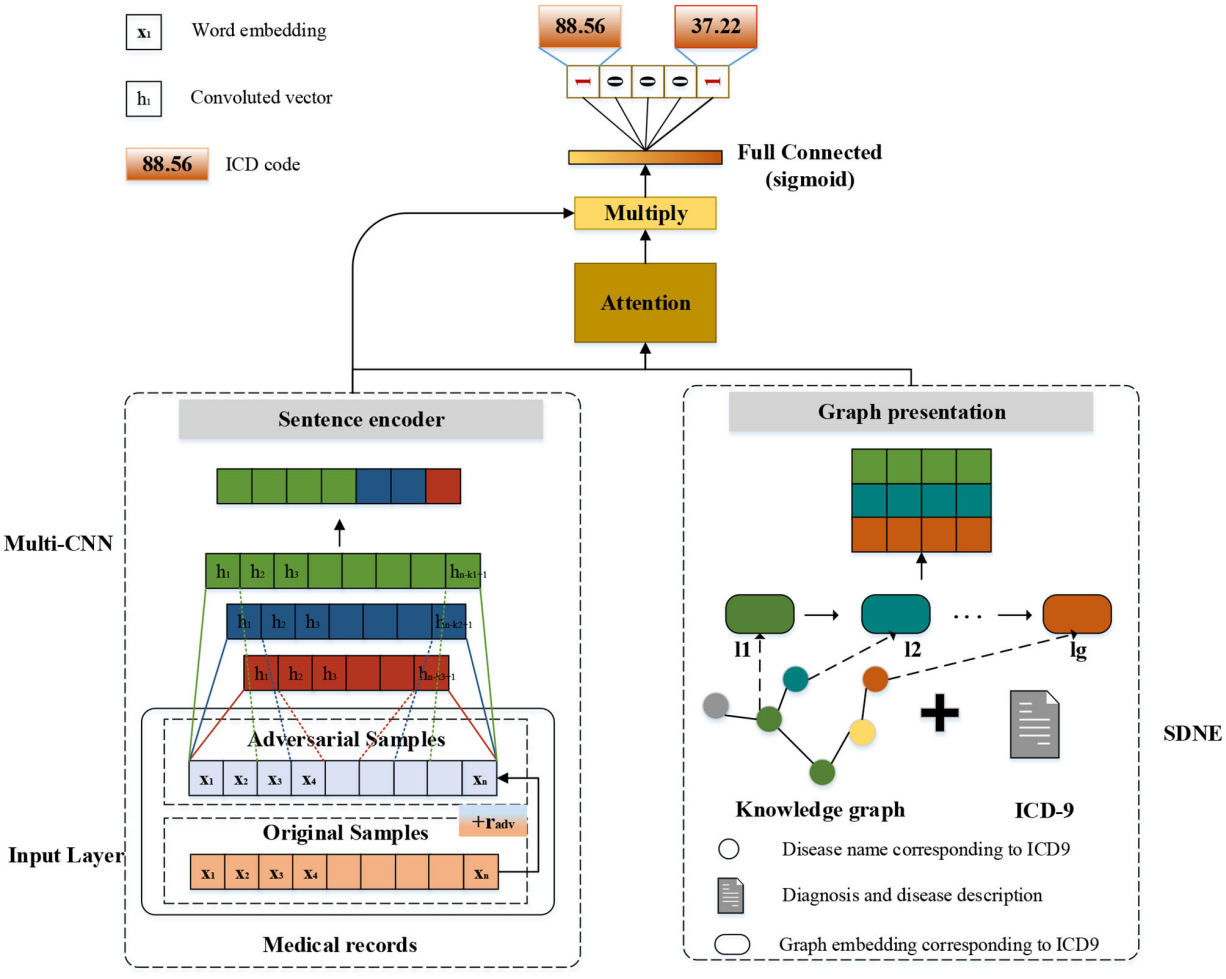
The overview of G_Coder.

The modular method adopted in this study differs from the researchers used earlier. Figure 3 shows an overview of our approach named G_Coder. The proposed approach mainly consists of four modules, which mainly contain Multi-CNN, Graph Presentation, Attentional Matching, and Adversarial Learning.

#### Input Layer

Considering that the pre-trained word vectors in the medical field are not yet perfect and the experimental data in this study are very long texts, the word embeddings were initialized randomly. Leveraging a token sequence *x* = {*x*_1_, *x*_2_, *x*_3_…, *x*_*n*_} as input, where *n* denotes the sequence length. Assuming that the matrix *W* denotes the word embedding matrix, and *W* = {*w*_1_, *w*_2_, *w*_3_, …, *w*_*v*_}∈ ℝ^*v*×*d*^, where *v* represents the size of total vocabulary and *d* represents the token dimension. The vocabulary is obtained by pre-processing the MIMIC-III clinical text. A token *x*_*i*_ will correspond to a vector *w*_*j*_ by looking up *W*. The final input of the model is a matrix *X* ∈ ℝ^*n*×*d*^.

#### Multi-CNN

As can be seen from Figure 3, the structure of Multi-CNN is used to encode the input matrix *X*. Multi-CNN is a combination of multiple CNNs and MaxPooling. CNN is a kind of neural network algorithm that has successfully been applied to computer vision. MaxPooling reduces the dimension of the feature map, and effectively reduces the parameters required for subsequent layers. Besides, it magnifies the receptive field.

Multiple kernels of different sizes are used to extract key information in the sentence, which inspired by Kim (2014) who applied Text-CNN to the text classification task. Multi-CNN is used to better capture the local correlations. Assuming we have filters *f*_1_, *f*_2_, …, *f*_*m*_ where m denotes the filter number. Each kernel size of filters denotes as *k*_1_, *k*_2_, ., *k*_*m*_. The convolutional procedure can be formalized as formula (1),

(1) $$\begin{array}{r} H_{1}=g({W_{c1}}^{*}x_{i:i+k-1}+b_{c1})\\ H_{m}=g({W_{cm}}^{*}x_{i:i+k-1}+b_{cm}) \end{array}$$

where * denotes the convolution operator, *g* is an element-wise non-linear transformation, *W*_*cm*_ is weight parameter and *b*_*cm*_ is the bias. Assuming that *H*_*m*_ = {h_1_, h_2_, h_3_, …, h_n−k+1_} is the output of m-th CNN and *Hm*′ is the output of m-th MaxPooling. The result of Multi-CNN is $H^{\prime }=\left[H1^{\prime }⊕H2^{\prime }⊕\ldots ⊕Hm^{\prime }\right]\in {\mathbb{R}}^{\overset{m}{\underset{1}{\sum }}dt}$, where ⊕ denotes concatenation operator and *d*_*t*_ denotes the dimension of *Ht*′.

#### Graph Presentation

In this study, we mainly adopt SDNE (Structural Deep Network Embedding) for medical knowledge graph node embedding. First-order proximity and second-order proximity are two crucial definitions in SDNE. The first-order proximity is used to describe the local similarity between paired nodes in the graph. If there are no directly connected edges, the first-order proximity is 0. The second-order proximity measures the similarity of their neighbor sets between two nodes. The optimization goal of SDNE is shown in formulas (2–4):

(2) $$\begin{array}{r} L_{1st}=\overset{n_{d}}{\underset{i,j=1}{\sum }}{s_{i,j}||r_{i}-r_{j}||}_{2}^{2} \end{array}$$

Each *s*_*i*_ contains the neighbor structure information of the i-th node. The letter *r* denotes the vector representation of each node. Where *n*_*d*_ denotes the number of neighbors at nodes *i*.

(3) $$\begin{array}{r} L_{2st}=\overset{n_{d}}{\underset{i=1}{\sum }}{\left||\hat{s_{i}}-s_{i}|\right|}_{2}^{2} \end{array}$$

(4) $$\begin{array}{r} L=L_{1st}+{\alpha L}_{2st}+\beta L_{reg} \end{array}$$

*L*_1*st*_ makes the embedding vectors corresponding to the two adjacent nodes in the graph close in the hidden space. *L*_*reg*_ is a regularization constraint, α is a parameter that controls the first- proximity loss, and β is a parameter that controls the regularization constraint. After SDNE, each node gets its own vector representation in the hidden space. Assuming that the matrix *y*_*g*_ is the result linked to ICD-9 of SDNE, which $\in {\mathbb{R}}^{l_{g}\times d_{g}}$. Where *l*_*g*_ denotes the number of ICD-9 and *d*_*g*_ denotes the dimensions of each node.

#### Attentional Matching

Human coders usually look for the most critical part of the medical record (Such as symptoms, complications, etc.) to determine the final coding result. In this task, we need to refine the text that most relevant to the ICD information and give higher weight. For the above reasons, we apply the attention mechanism. A benefit is that it selects the segments from the text that are most relevant to each predicted label. The specific algorithm details are shown in Table 2. It obtained the clinical text representation vector *H*′ through preprocessing and Multi-CNN, and at the same time obtained the ICD coded representation *y*_*g*_ using the knowledge graph embedding results. A linear transformation was performed on the code representation to obtain the final code representation *D*, which has the same dimensions as the number of codes. The text representation *H*′ and label representation *D* are used to calculate the weight *a*_*i*_ of the relationship between each label and each segment of the text. Finally, the text *H*′ and weight *a*_*i*_ are used to weight the average of each part of the text to obtain the final clinical text representation *v*_*i*_.

**Table 2:** The algorithm details of attentional matching.

**Algorithm1: Attentional matching**
For each ***H*′** from Multi-CNN: 1. Calculate label representation vector ***D***; _***D* = (*W***_***g***_***yg***_**+*b*)** 2. The ***a***_***i***_ Measures how informative each n-gram is for the i-th label.; $a_{i}=SoftMax\left({H^{\prime }}^{T}D_{i}\right),i=1,2,3\ldots ,l_{g}$ 3. Calculate the weighted average ***v***_***i***_ of the rows in ***H*′** forming a vector representation of the clinic text for the i-th label; $v_{i}=\;a_{i}H^{\prime }$

The results in Table 2 can be summarized as follows:

(5) $$\begin{array}{r} A=SoftMax\left({{{H^{{}^{\prime }}}^{T}W}_{g}y}_{g}\right),A=\left[a_{1},a_{2},\ldots ,a_{\mathrm{lg}}\right] \end{array}$$

(6) $$\begin{array}{r} V=AH^{{}^{\prime }},A\;=\;\left[v_{1},v_{2},\ldots ,v_{\mathrm{lg}}\right] \end{array}$$

Where $SoftMax\left(x\right)=\;\frac{\exp\left(x_{i}\right)}{\underset{i}{\sum }\exp\left(x_{i}\right)}$, and exp is an exponential function with natural constant e as a base. The matrix $W_{g}\;\in {\mathbb{R}}^{d_{g}\times l_{g}}$ is the weight parameter. And *A* denotes attention weights for each pair of an ICD code and the text. The letter $V\in {\mathbb{R}}^{l_{g}\times l_{g}}$ denotes the output of the attention. The concrete example can be found in **Table 7**.

#### Adversarial Learning

We apply FGM (fast gradient method) to reconcile the different writing styles of doctors and increase training samples (Miyato et al., 2017). The basic idea is: The writing of medical records follows the writing standards, but also contains different writing styles. Adversarial learning weakens the influence of writing style. The purpose of adversarial training is that the model will work steadily even if there are large differences in doctor writing styles. FGM uses a first-order Taylor expansion on the adversarial objective function to approximate to maximize the error output by the model, which is equivalent to using a single-step gradient descent method with a step size of ϵ to find the adversarial samples. The specific algorithm details are shown in Table 3. It calculates the gradient *g* of the clinic text embedding *X* after forward propagation and then back propagation. The gradient is used to calculate the perturbation *r*_*adv*_ added to *X*. After such a process, *X*_*adv*_ is an automatically generated adversarial sample. It uses the adversarial samples to calculate together with the original samples, increasing the number of samples, while mimicking the writing style of different doctors.

**Table 3:** The algorithm details of Adversarial Learning.

**Algorithm 2: Adversarial learning**
For each ***X*** in training samples: 1. Calculate the forward loss of ***X*** and get the gradient ***g*** by back propagation; ***g*=∇**_***X***_***L*(θ,*X*,*Y*)** 2. Calculate ***r***_***adv***_ according to the gradient of the embedding matrix ***X*** and add it to the current embedding, which is equivalent to ***X*+*r***_***adv***_; ***r***_***adv***_**=ϵ∙*g*/|**|***g***|**|2** ***X***_***adv***_**=*X*+*r***_***adv***_ 3. Calculate the forward loss of ***X***_***adv***_, backpropagate to obtain the gradient of the confrontation, and add to the gradient of step 1; 4. Restore embedding to the value at step 1; 5. Update the parameters according to the gradient of step 3.

The goals of adversarial learning are as follows:

(7) $$\underset{\theta }{\min}E\;\left(X,Y\right)\sim D[\underset{r_{adv}\varepsilon R}{\max}(L(\theta ,X_{adv},Y))]\;$$

The formula (7) is divided into two parts, one is the maximization of the internal loss function, and the other is the minimization of the external risk. In the internal max, *L* is the defined loss function, *D* is the perturbation of input samples, and *R* is the space for a perturbation. The goal of adversarial learning is to find the amount of perturbation that makes the most judgment errors. For the above attacks, the most robust model parameters are found. After further optimizing the model parameters, the expected value of the entire data distribution is still minimal.

#### Output Layer

We compute a probability for label vector $Ŷ\in {\mathbb{R}}^{l_{g}}$ using full connection layer and a sigmoid transformation by the output of attention representation *V*:

(8) $$\begin{array}{r} \hat{Y}=\sigma \left(W_{o}V\right) \end{array}$$

Where $W_{o}\in {\mathbb{R}}^{l_{g}\times l_{g}}$ is learnable weights of output layer and $\sigma \left(x\right)=\frac{1}{1+exp\left(-x\right)}$. The whole learning process minimizes the binary cross-entropy loss (9) of prediction probability $\hat{Y_{i}}$ and the target *Y*_*i*_ ∈ (0, 1). The label *i* is selected when $\hat{Y_{i}}$ is >0.5.

(9) $$\begin{array}{r} L\left(\theta ,X,Y\right)=-\overset{l_{g}}{\underset{i=1}{\sum }}Y_{i}\log\left(\hat{Y_{i}}\right)+\left(1-Y_{i}\right)\log\left(1-\hat{Y_{i}}\right)+{\lambda ||\gamma ||}_{2}^{2} \end{array}$$

Where *X* denotes the input word sequence, λ is the L2 regularization hyperparameter. And θ denotes all the parameters. We utilize the back-propagation algorithm and Adam optimizer (Kingma and Ba, 2014) to train the model.

## Experiments

### Experimental Settings

A majority of codes are only assigned to too few medical records. Since the top 50 common ICD-9 codes covered 93.6% of the all dataset, we pick 50 most frequent codes to carry out the experiment while considering that our method can readily be extended to more codes as long as sufficient training data is available. The experimental dataset using top-50 codes has a total of 46,552 discharge summaries, which has 43,000 discharge summaries for training, 1,800 for validation, and 1,752 for the test. In this experiment, the settings are shown in Table 4. The token dimension *d* is 100; the knowledge graph embedding size *d*_*g*_ is 128; the out-channel size *d*_*f*_ of a filter in the Multi-CNN layer is 50; the learning rate *lr* is 0.001; the L2 regularization hyperparameter λ is 0.00001; the max length of each medical record is 1,800; the mini-batch size is 16 and the dropout rate *dp* is 0.4. We used three filters and the kernel size of filters is 4,5,6.

**Table 4:** The hyperparameter settings of the experiment.

**Hyperparameter**	**Value**
*d*	100
*d*_*g*_	128
*d*_*f*_	50
*lr*	0.001
*dp*	0.4
λ	0.00001
Filters size	{4,5,6}

### Evaluation Metrics

This task can be regarded as a multi-label classification problem. Therefore, we evaluate the method by *micro*−*F*1 and AUC (Area under the curve). The *micro*−*F*1 is harmonic mean that calculated from *Precision* and *Recall*. All evaluation matrixes are calculated as follows:

(10) $$\begin{array}{r} Precision=\;\frac{\overset{n}{\underset{i=1}{\sum }}{TP}_{i}}{\overset{n}{\underset{i=1}{\sum }}{TP}_{i}+\overset{n}{\underset{i=1}{\sum }}{FP}_{i}} \end{array}$$

(11) $$\begin{array}{r} Recall=\;\frac{\overset{n}{\underset{i=1}{\sum }}{TP}_{i}}{\overset{n}{\underset{i=1}{\sum }}{TP}_{i}+\overset{n}{\underset{i=1}{\sum }}{FN}_{i}} \end{array}$$

(12) $$\begin{array}{r} micro-F1=\frac{2\times Recall\times Precision\;}{Precision+Recall} \end{array}$$

In these formulas, *TP*_*i*_ is the set of ground truth labels of each class, *n* is the number of samples, *FN*_*i*_ is the number of positive classes predicted as negative classes and *FP*_*i*_ is the number of negative classes predicted as positive classes. AUC is mainly used to evaluate the ranking ability of the current model. The higher the AUC, the better the ranking ability of the model. When the prediction probability values of all positive samples are higher than the negative samples, the AUC of the model is 1.

### Results

#### Model Comparison

This section illustrates the performance of our approach. The experimental results of the top-50 codes show in Table 5, which show that our work has improved on previous work. CNN-Att is the baseline model for this experiment, which uses CNN to encode text. MultiResCNN has achieved the state-of-the-art results on the MIMIC-III datasets using unstructured text. Besides, their work is based on CAML and the model is improved. It mainly consists of a multi-filter convolutional layer and residual convolutional layer for multi-label classification. C-LSTM-Att applied LSTM-based language models to encode clinical notes and ICD codes and applied an attention method to solve the mismatch between clinical notes and codes. They focused on predicting the 50 codes that have the top frequencies for the medical records in the MIMIC-III dataset just like us.

**Table 5:** The experimental results of the top-50 codes.

**Method**	**micro-F1**	**micro-AUC**	**P@5**
CNN-Att	0.625	0.907	0.620
C-LSTM-Att Shi et al. (2017)	0.532	0.900	-
CAML Mullenbach et al. (2018)	0.614	0.909	0.609
DR-CAML Mullenbach et al. (2018)	0.633	0.916	0.618
MultiResCNN Li and Yu (2019)	0.673	0.928	0.641
No-knowledge-graph	0.670	0.923	0.637
No-adversarial-learning	0.681	0.929	0.647
**G_Coder**	**0.692**	**0.933**	**0.653**

*Bold represent the current model result*.

Comparing our model with existing work for automatic ICD coding. As shown in Table 5, the conclusions are as follow:

1. G_Coder obtains better results in the micro-AUC, micro-F1, and P@5. Compared with the state-of-the-art model MultiResCNN, G_Coder improves the micro-AUC by 0.005, the micro-F1 by 0.019, the P@5 by 0.012. P@5 measures the ability of the method to return the top 5 high-confidence subsets of codes. Our approach achieves relatively high precision of the five most confident predictions, on average 3.3 are correct.
2. CNN-based models are more suitable for this task. LSTM pay more attention to capture long sequence features, and cannot extract important local features from noise text. Simultaneously, the length of the medical record text makes the recurrent neural network have extremely high requirements for machine performance in this task. In contrast, it can be seen from the model construction that CNN can better extract long text features, and multilayer CNN with different convolution kernels can better capture local correlation.
3. The attention mechanism is essential. Each model utilizes the attention mechanism, which shows that the mechanism accurately highlights the information related to ICD in the text. The following content will prove the value of the knowledge graph and adversarial learning in this task.

#### Ablation Study

To gain more insight, the ablation study applied to verify the effectiveness of the adversarial learning and knowledge graph. To evaluate each module, we perform single variable experiments. The comparisons of the No-one module with the full model are given in Table 6. We remove one module from the full model without changing other modules and denote such a baseline by No-X. To evaluate them, we compared with the two configurations: (1) No- knowledge-graph, which removes the graph presentations and directly uses a randomly initialized vector as final representations of codes information; (2) No- adversarial-learning, which removes the adversarial learning form full model.

**Table 6:** The result of universality study.

**Method**	**micro-F1**	**micro-AUC**	**P@5**
CNN-Att	0.625	0.907	0.620
CNN-Att- graph	**0.651**	**0.920**	0.619

*Bold represent the best results*.

It can see from Table 6 that our full model obtains better results in all evaluation matrix. Compared with the full model, No-knowledge-graph dropped the micro-AUC from 0.933 to 0.923, the micro-F1 from 0.692 to 0.670, the P@5 from 0.653 to 0.637. At the same time, No-adversarial**-**learning dropped the micro-AUC from 0.933 to 0.929, the micro-F1 from 0.692 to 0.681, the P@5 from 0.653 to 0.647. The above results show that the knowledge graph-based method can add clinical experience to make the results better. And adversarial learning generates adversarial samples through perturbation factors to enhance the generalization ability of the model on the test set. From the results we have obtained, one can conclude that the combination of data-driven and medical knowledge can enhance the precision of ICD automatic coding.

#### Universality Study

To prove that the knowledge graph is universal in this task. We design the experiment, which is to add a knowledge graph to the basic baseline model and compare it with the baseline model.

According to the experimental results in Table 6, it can be seen that the knowledge graph not only performs well in G_Coder but also can be extended to other model structures. The knowledge graph improves the micro-F1 of the baseline model by 2.6%. This shows that the knowledge graph is universal and can be flexibly grafted into other model structures.

#### Evaluation of Interpretability

We use two methods to verify the interpretability. The first is an intuitive method that attention extracts keywords and displays the correlation between the code and the evidence. Examples can be found in Table 7. It can be seen from which words the basis of coding comes from. Taking 584.9 as an example, there is an information overlap between “acute renewal failure, unspecified” and “with acute renewal failure secondary” in clinical texts.

**Table 7:** Presentation of clinical text fragments and their corresponding ICD codes (The bold part indicates the highest weight).

**ICD-9 codes and description**	**The highest weighted part**
584.9 Acute renal failure, unspecified	…support **with acute renal failure secondary** to the prolong hypertension…
410.71 Acute myocardial infarction, subendocardial infarction, initial episode of care	…the patient experienced **right ventricular failure and went** back on bypass with drug manipulations…
414.01 Coronary atherosclerosis of native coronary artery	…with a right heart bypass cannulation in place. The patient **was profoundly hypoxic and acidotic**. …
428.0 Congestive heart failure, unspecified	…He also had lactic **acidosis and congestive heart failure**. The hypernatremia. …

The second is a quantitative method where doctors judge the results of attention distribution. A clinical medical record was randomly selected, and segments were extracted based on the results of its attention. We select 5-words in this setting to emulate a span of attention over words likely to be given by a human reader. Since the segment may overlap, the most important 5-words were extracted according to attention weight. As can be seen from Table 8, the score is divided into two stages, one is high weight, that is >0.8, and the other is <0.8. In a total of 100 segments, there are 16 with a weight >0.8 and 84 with a weight <0.8. According to the evaluation results of human coders, 10 of the high weights are correct, and the remaining correct number is 60.

**Table 8:** The result of the evaluation of interpretability.

**Type**	**Total**	**Correct**	**Accuracy**
High weight (weight ≥ 0.8)	16	10	0.625
Others (weight <0.8)	84	60	0.714

## Conclusions and Discussions

### Conclusions

Inspired by the structure of graphs that can model the relationships and knowledge between all things in the world, we think the graph structure can connect the parts of the data in this task and create a knowledge graph using medical-related data from the Freebase database. At the same time, the development of deep learning has also allowed further development of natural language processing such as automatic coding and text classification. In this paper, we propose a new explainable method for automatic ICD coding. The result of the micro-F1 score of 50 most frequent codes is 69.2%, which outperforms all the other models especially when raw clinical text data is used as input features to the prediction models.

The experimental evaluation of the MIMIC-III dataset shows the following points. First, we combined deep learning with knowledge graphs in ICD coding tasks. The medical knowledge graph supervises the coding process as a teacher. At the same time, we apply the SDNE algorithm to encode each entity of the knowledge graph and link it to the ICD-9 code. The Multi-CNN algorithm is utilized to encode long text information of MIMIC-III data. In the attention mechanism, we combine the two mentioned above to identify the segments of text that are most relevant to each ICD-9 code. Finally, we generate adversarial samples through adversarial training and send the samples to the training along with the original samples. It can weaken the influence of writing style and make model more stable. Moreover, in the ablation study and universality study, we use the single variable rule to verify the importance of adversarial learning and knowledge graph. The results prove that the knowledge graph can be flexibly grafted into the model structure to help understand the terminology. Two methods are used to verify the interpretability of the method. It is confirmed that this method is based on the important basis in the clinical text for ICD coding. G_Coder has a higher accuracy rate than the other method. And before the coder works, G_Coder can perform ICD pre-selection to save time for whole encoding work.

### Discussions

The major limitation of this work is that it does not perform well on infrequent codes. To achieve fully automatic coding, infrequent coding has to be considered. And we hold that the method can readily be extended to more codes as long as sufficient training data is available. In addition, the new ICD version should also be considered, such as ICD10, ICD11, etc. ICD classification is a disease classification directory with hierarchical relationship. The structure of ICD is also a direction worth considering.

## Data Availability Statement

Publicly available datasets were analyzed in this study. This data can be found here: http://freebase.gstore-pku.com/, https://mimic.physionet.org/, https://developers.google.com/freebase.

## Author Contributions

WY and FT provided total research ideas designed the experiments. WY performed the experiments and wrote the first draft of the manuscript. LH and LC guided the experiment as experts and analyzed the results. FT contributed to the High-performance experimental equipment. WY and QX contributed to manuscript revision, reading, and approving the submitted version. All authors contributed to the article and approved the submitted version.

## Conflict of Interest

The authors declare that the research was conducted in the absence of any commercial or financial relationships that could be construed as a potential conflict of interest.
